# Human Angiostrongyliasis Outbreak in Dali, China

**DOI:** 10.1371/journal.pntd.0000520

**Published:** 2009-09-22

**Authors:** Shan Lv, Yi Zhang, Shao-Rong Chen, Li-Bo Wang, Wen Fang, Feng Chen, Jin-Yong Jiang, Yuan-Lin Li, Zun-Wei Du, Xiao-Nong Zhou

**Affiliations:** 1 National Institute of Parasitic Diseases, Chinese Center for Disease Control and Prevention, Shanghai, People's Republic of China; 2 Institute of Research and Control of Schistosomiasis in Dali Prefecture, Dali, People's Republic of China; 3 Yunnan Institute of Parasitic Diseases, Puer, People's Republic of China; PUCRS University, Brazil

## Abstract

**Background:**

Several angiostrongyliasis outbreaks have been reported in recent years but the disease continues to be neglected in public health circles. We describe an outbreak in Dali, southwest China in order to highlight some key problems for the control of this helminth infection.

**Methodology/Principal Findings:**

All available medical records of suspected angiostrongyliasis patients visiting hospitals in Dali in the period 1 October 2007–31 March 2008 were reviewed, and tentative diagnoses of varying strengths were reached according to given sets of criteria. Snails collected from local markets, restaurants and natural habitats were also screened for the presence of *Angiostrongylus cantonensis*. A total of 33 patients met criteria for infection, and 11 among them were classified as clinically confirmed. An additional eight patients were identified through a surveillance system put in operation in response to the outbreak. The epidemic lasted for 8 months with its peak in February 2008. Of the 33 patients, 97.0% complained of severe headache. 84.8% patients had high eosinophil cell counts either in the peripheral blood or in cerebrospinal fluid (CSF). Three-quarters of the patients were treated with a combination of albendazole and corticosteroids, resulting in significantly improved overall conditions. Twenty-two patients reported the consumption of raw or undercooked snails prior to the onset of the symptoms, and approximately 1.0% of the *Pomacea canaliculata* snails on sale were found to be infected with *A. cantonensis*. The snails were also found in certain habitats around Dali but no parasites were detected in these populations.

**Conclusions/Significance:**

The import and sale of infected *P. canaliculata* is the likely trigger for this angiostrongyliasis outbreak. Awareness of angiostrongyliasis must be raised, and standardized diagnosis and treatment are needed in order to provide clinicians with a guide to address this disease. Health education campaigns could limit the risk, and a hospital-based surveillance system should be established in order to detect future outbreaks.

## Author Summary

Angiostrongyliasis, caused by the rat lungworm *Angiostrongylus cantonensis,* is a potentially fatal food-borne disease. It is endemic in parts of Southeast Asia, the Pacific Islands, Australia, and the Caribbean. Outbreaks have become increasingly common in China due to the spread of efficient intermediate host snails, most notably *Pomacea canaliculata*. However, infections are difficult to detect since the disease has a rather long incubation period and few diagnostic clinical symptoms. Reliable diagnostic tests are not widely available. The described angiostrongyliasis epidemic in Dali, China lasted for eight months. Only 11 of a total of 33 suspected patients were clinically confirmed based on a set of diagnostic criteria. Our results demonstrate that the rapid and correct diagnosis of the index patient is crucial to adequately respond to an epidemic, and a set of standardized diagnostic procedures is needed to guide clinicians. Integrated control and management measures including health education, clinical guidelines and a hospital-based surveillance system, should be implemented in areas where snails are a popular food item.

## Introduction

Angiostrongyliasis caused by *Angiostrongylus cantonensis* is a potentially fatal parasitic disease. Humans are infected through several ways as its life cycle involves different edible intermediate host mollusks and a range of paratenic hosts which also pose threats [1]. In China and Southeast Asia, consumption of raw or undercooked snails, mainly *Pomacea canaliculata*, *Achatina fulica* and *Pila* spp., is the primary route of infection [2]–[4]. In the Caribbean, contaminated vegetables and condiments have been implicated, e.g. in an outbreak in Jamaica [5]. Monitor lizard is considered as the main source of infection in India and Sri Lanka [6],[7] while freshwater shrimps, fish and crabs are a suspected way of infection in the Pacific Islands [8],[9]. Angiostrongyliasis has been widely spread in the wake of biological invasion. *Rattus norvegicus* and *A. fulica* are two top invasive species and *A. cantonensis* is thought to have trailed the two animals in establishing itself throughout the tropics [10]. Indeed, many outbreaks and sporadic cases were attributed to *A. fulica* [4], [11]–[13] or happened only after the invasion of the two species [14]. Recently, the snail rapidly spread into Brazil [15], and only subsequently, *A. cantonensis* had been found to naturally infect local mollusks [16]. In addition to *A. fulica*, another invasive snail species, i.e., *P. canaliculata*, has facilitated the emergence of angiostrongyliasis in Asia in the past decade and has since become the primary vector in this region [2],[17].

Over 2,800 cases have already been reported from more than 30 countries [18] but this figure might well be a small fraction of the real number. There are sporadic cases which are likely to go undetected due to low awareness among the medical community and a lack of diagnostic symptoms and readily available tests. Some patients also only experience transient or mild manifestations [10],[19]. Angiostrongyliasis is not common and is usually clustered in certain population segments. Recently, cases in returning travelers from non-endemic regions have been reported [20]–[25]. Others include local people with special dietary habits or using traditional medicine [26].

Here, we describe an outbreak in Dali in Yunnan province, southwest China in order to highlight this neglected disease and the challenges involved in its diagnosis, surveillance and control.

## Materials and Methods

### Ethics statement

The investigation had been approved by the Academic Board (ethics committee) of the National Institute of Parasitic Diseases, Chinese Center for Disease Control and Prevention in Shanghai (ref. no. 2006111201). All participants were informed about the study procedures and gave their written informed consent or, in the case of illiterates, oral approval.

### Retrospective survey and establishment of surveillance system

In response to an unusually high number of suspicious clinical manifestations, all medical records of suspected angiostrongyliasis patients visiting three major hospitals in Dali, namely People's Hospital of Dali Prefecture, Dali First People's Hospital, Affiliated Hospital of Dali Medicine College, and a specialized local medical center (Institute of Research and Control of Schistosomiasis in Dali Prefecture) between 1 October 2007 and 31 March 2008 were reviewed. The extracted data included demographic information, onset of illness, hospitalization date, clinical manifestations, examination and laboratory test results, disease progression, and treatment regimens and outcomes. The patients, including discharged patients, were then traced and interviewed regarding potential exposure (e.g. consumption of raw or undercooked snails, fish, shrimps and crabs within the past one month), the place and date of eating these food items, and the number of persons who shared the same food.

In order to follow the progress of the epidemic, a temporary hospital-based surveillance system was established to collect information on new cases from 1 April 2008 onwards. Through this system, clinicians reported suspected patients according to a given set of diagnostic criteria.

### Diagnostic criteria

Patients were stratified into three groups according to the tentative diagnostic criteria of angiostrongyliasis published by the Ministry of Health in 2006. The three classes were suspected, clinically diagnosed and parasitologically diagnosed cases. The diagnostic criteria are based on available evidence from clinical practice:

1. Eating history: recently (within one month) ate raw or undercooked snails or other potentially infective food items such as slugs as medicine, raw or undercooked freshwater fish, shrimps, crabs, frogs and snakes.
2. Clinical manifestations: presenting with at least one of the following symptoms: severe headache, nausea and vomiting, visual disturbances, photophobia, nuchal rigidity, hyperesthesia, and paresthesia.
3. An elevated count of eosinophils (>500 cells/µL) in peripheral blood.
4. An elevated count of eosinophils (>10 cells/µL) in cerebrospinal fluid.
5. Sero-positive for specific *A. cantonensis* antigens or the corresponding antibody.
6. Presence of *A. cantonensis* larvae in cerebrospinal fluid, anterior chamber, vitreous cavity, or subretinal space; or presence of larvae in sections of the brain and spinal cord, or worms in pulmonary arteries or the heart.

Suspected cases were those meeting criteria 1 and 2, or 1 and 5, or 2 and 3, or 2 and 4. Clinical diagnosis required meeting the criteria 1, 2, 3 and 4. Parasitologically diagnosed cases were those clinical cases where *A. cantonensis* was discovered as stipulated by criterion 6.

### Additional investigations

In order to understand the local prevalence of *A. cantonensis* in snails marketed for human consumption, *P. canaliculata* and *Cipangopaludina chinensis* snails were collected in markets and restaurants mentioned by study participants. A week-long surveillance of the snails on sale was conducted from 26–31 March 2008. *A. cantonensis* infections in *P. canaliculata* were diagnosed using the “lung examination” method described by Liu and co-workers [27]. Negative snails were examined in batches of five using the artificial digestion method [28]. *C. chinensis* were examined by artificial digestion only. In addition, the sources of snails available on markets and in restaurants were investigated. Snails including *P. canaliculata* and *C. chinensis* collected from natural habitats were also examined using the same techniques.

### Data analyses

All data were entered in a central database using EpiInfo version 3.5 (Centers for Disease Control and Prevention, Atlanta, USA). The epidemic trend of angiostrongyliasis in Dali between 1 October 2007 and 31 March 2008 was described using bar graphs with a two-week interval. Both the onset of manifestations and the date of hospital admission for each patient are shown. Demographic information, i.e. sex, age and ethnic group, were investigated, and the proportion of suspected sources of infection (e.g. snails, fish and shrimps, unknown) calculated and clinical manifestations summarized.

The dynamic relationship between eosinophil counts in peripheral blood and CSF was analyzed using available paired data of all patients collected before treatment and over the course of the intervention to describe their co-dynamic pattern. Increased eosinophil cell counts in peripheral blood were defined as those with more than 500 cells per µL, and increased eosinophil counts in CSF were those with more than 10 cells per µL. Since the date of collection of blood and CSF samples might differ, only data originating from samples collected within an interval of less than 3 days were considered.

The treatment efficacy of the standard combination therapy involving albendazole and dexamethasone was assessed by following changes in eosinophil cell counts in the peripheral blood. Data on eosinophils were collected before onset of any treatment which might affect eosinophil cell numbers and again during and after combination treatment. The effect of illness duration (the duration of chief complaints, i.e., headache for 32 patients, numbness and muscle weakness in limbs for one patient) and the period of drug treatment were also considered.

## Results

### Epidemiology

**Figure 1:**
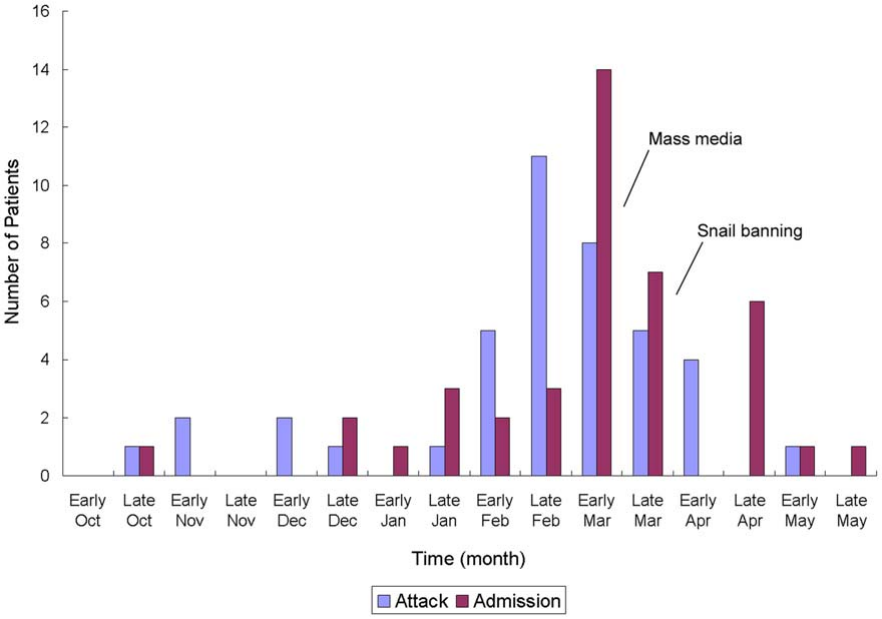
Course of the angiostrongyliasis epidemic in Dali from October 2007–May 2008. “Attack” and “Admission” designate the onset of symptoms and admission to hospital, respectively. “Mass media” indicates propagation of information on angiostrongyliasis through mass media and was initially performed in mid-March 2008. “Snail banning” is the prohibition of snail sales on markets since 1 April 2008.

Starting in mid-October 2007, the three major hospitals of Dali and a specialized medical center in the same city were visited by patients with eosinophilic meningitis. The last such patient was admitted to hospital in mid-May 2008. At this time, two public health interventions had been launched: information about angiostrongyliasis had been spread through mass media in mid-March, and the sale of live snails in markets had been banned since 1 April. The peak incidence was in late February and the peak of hospital admissions was in early March (Figure 1). None of the suspected angiostrongyliasis cases which were characterized by significantly elevated eosinophil cell counts and typical manifestations had been parasitologically confirmed. Of 36 patients included in the retrospective survey, 3 were excluded due to lack of eating history of raw snails or other potential infective food items. Among 33 cases who met the diagnostic criteria set by the Ministry of Health, 11 could be classified as clinically diagnosed cases and 22 were suspected cases (Table S1). Another 8 cases (one suspected and seven clinical cases) were picked up by the hospital-based surveillance system between 1 April and 30 September 2008.

Of the 33 patients included in this retrospective study, 18 were female (Table 1). They were aged 12–72 years and mainly belonged to the Bai ethnic minority (*n* = 19) or were Han Chinese (*n* = 13). All patients were locals except three who lived in adjacent counties but had consumed the potentially infectious food items in Dali city. Consumption of raw or undercooked snails in the past one month was reported by 22 study participants, and eating raw fish or unknown food items possibly related to angiostrongyliasis equally accounted for the remaining cases. The study participants mentioned a range of locations and time points where they had consumed potentially infective food: four patients ate snails obtained in local markets in private settings, six pointed out the same restaurant but had frequented it at different dates, and others had dined at several other restaurants. Most individuals had consumed the suspicious food in early February 2008 around the Spring Festival/traditional Chinese New Year.

**Table 1:** Characteristics of 33 patients involved in the angiostrongyliasis epidemic in Dali in 2007–2008.

Characteristics	Patients (N = 33)
**Demographic information**	
Sex, *n* (%)	
Male	15 (45.5)
Female	18 (54.5)
Age, median (range)	35 (12–72)
Ethnic group-no., *n* (%)	
Bai	19 (57.6)
Han	13 (39.4)
Other	1 (3.0)
**Reported eating history (** ***n*** ** [%])**	
Snails	22 (66.7)
Fish/shrimps	6 (18.2)
Unknown	5 (15.1)
**Symptoms and signs (** ***n*** ** [%])**	
Headache	32 (97.0)
Nausea and vomiting	13 (39.4)
Nuchal rigidity	6 (18.2)
Muscle weakness	5 (15.1)
Fever (axillary temperature >37.8°C)	4 (12.1)
Paresthesias	3 (9.1)
Muscle pain	3 (9.1)
Visual disturbance	2 (6.1)

### Clinical characteristics

**Figure 2:**
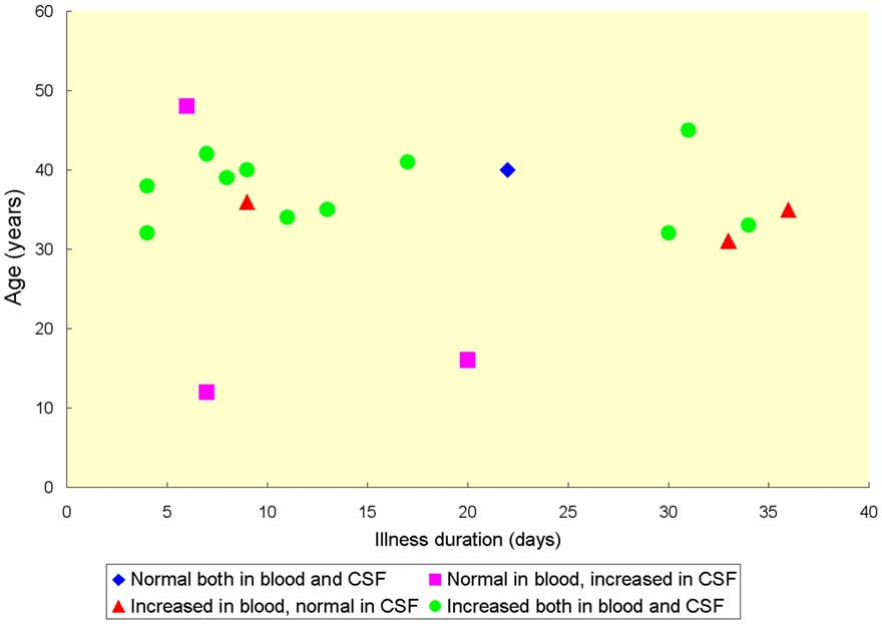
Eosinophil counts in peripheral blood and CSF before treatment, stratified by illness duration and age. An increased level of eosinophil cell counts in blood is defined as a relative count of more than >500 cells/µL and an increased eosinophil level in CSF is defined as a count of more than >10 cells/µL. The data of the 18 patients were collected before treatment. The collection date of blood and CSF samples from the same patient may differ up to 3 days.

The mean estimated incubation period among the 33 patients was 16 days with a range of 3–50 days. Headache was reported by 97.0% of all patients when they were admitted to hospital (Table 1). One case only suffered from numbness and muscle weakness of the left limbs. Nausea and vomiting were reported by 39.4% of the cases. Fever was uncommon; most were afebrile during the entire duration of their illness. Thirty patients received lumbar punctures and 18.2% of the patients suffered from high intracranial pressure (>250 mmH_2_O). High eosinophil counts in peripheral blood and/or CSF were noted in 84.8% of the patients. Simultaneous cytological tests in peripheral blood and CSF before treatment were done in 18 patients, and 61.1% of them had elevated eosinophil counts in both blood and CSF (Figure 2). The three patients with increased counts of eosinophils in their CSF but normal blood values were the oldest and two youngest patients of the entire cohort. Two of the three patients with normal eosinophil counts in their CSF and increased counts in their blood had a disease history ≥33 days. Only one patient had normal eosinophil levels in both blood and CSF. Average glucose and chlorinate levels were 2.65±0.65 mmol/L and 122.59±10.00 mmol/L, respectively. Total protein varied between 189 mg/L and 3857 mg/L with a median of 753 mg/L. Differential serological testing was performed in 20 patients (schistosomiasis, trichinellosis, cysticercosis, echinococcosis) but only five patients were tested for *A. cantonensis*-specific antibody, among whom four tested positive.

### Treatment

A combination of albendazole (200–400 mg, tid) and dexamethasone (5–20 mg/day) was employed to treat 26 study participants. In addition, patients suffering from high intracranial pressure were treated with mannitol (20%; 250 ml/day). The other patients were treated with either albendazole or dexamethasone, each accompanied by mannitol, if necessary. Only one young female patient was readmitted to hospital due to relapse one month after she had been discharged. Continuous data reflecting the dynamics of key indicators, e.g. eosinophil counts, over the treatment period were only available for 7 patients. Eosinophil numbers in peripheral blood generally decreased after treatment but fluctuations were noted in two patients.

### Additional investigations

On 3 different days, *A. cantonensis* larvae were found in a total of five of the 503 *P. canaliculata* snails collected from local food markets. Samples from restaurants as well as *C. chinensis* samples were negative. Two separate *P. canaliculata* populations were discovered in the Dali area. The bigger habitat included Xihu Lake and its surroundings, upstream of Erhai Lake which is the biggest freshwater reservoir in the area. The second population was located in Heqing county in the vicinity of Dali city but none of the snails collected in these locations was found to be infected.

## Discussion

Dali city was considered non-endemic for angiostrongyliasis since neither human or animal cases nor infected specimens of known intermediate host mollusks were found in the area during the first national survey on angiostrongyliasis [29]. However, an extended outbreak occurred in this area due to importation of contaminated snail food. In previous angiostrongyliasis outbreaks in China both within and beyond the known endemic areas, most patients involved were found to have shared the same food at a single location [2],[5],[9],[17]. The described epidemic in Dali differed from this picture in several important aspects: patients consumed the potentially infective food at different places and over an extended period of time, suggesting a stable supply of contaminated food. Spring festival, the most important traditional Chinese holiday, arguably facilitated the epidemic since extended meals involving special dishes are a core feature of this holiday. The native *C. chinensis* is a traditional food item in many areas across China including Dali. The snail can readily be infected with *A. cantonensis* in the laboratory [30] and is an important source of infection in Taiwan [31],[32] but infected snails have rarely been detected in mainland China and no case of angiostrongyliasis in the country has ever been linked to this species. Since their population declined following environmental changes, *P. canaliculata* has increasingly been imported as a substitute, possibly also from places where *A. cantonensis* is endemic. Today, the majority of all outbreaks and sporadic cases in mainland China can be linked to this species [2],[33],[34].

None of the described angiostrongyliasis cases from Dali could be parasitologically confirmed but the clinical manifestations, laboratory data, eating histories as well as the presence of *A. cantonensis* in locally sold snails suggest the suspected infection. Indeed, the probability of finding *A. cantonensis* in patients is very low [2],[11],[19] and parasitological evidence is not considered mandatory for the identification of outbreaks since additional information obtainable in outbreak situations can help to reach the final decision [5],[17],[35]. This might explain the higher frequency of parasitological proof in sporadic cases than outbreak situations. We adopted the tentative diagnostic criteria of angiostrongyliasis set forth by the Chinese Ministry of Health. Based on them, our study population was categorized into 22 suspected and 11 clinically diagnosed cases. Several reasons drive this low approval rate of suspected cases. First, the criteria are strict compared to other clinic guidelines [5],[9],[11]. For example, clinical diagnosis requires elevated eosinophil numbers in both peripheral blood and CSF. However, in the present sample only 61.1% (11/18) showed consistency in eosinophil levels between peripheral blood and CSF before treatment, corroborating earlier observations [17]. Second, results from serologic tests are not considered for clinical diagnosis, leading to the classification as suspected cases of four seropositive patients. Although various immunological tests have been developed, none have been systematically evaluated and none are not widely and commercially available in China [18]. Last, important indicators might have been neglected due to low levels of awareness about angiostrongyliasis among medical staff. Thus, the development, evaluation and dissemination of serologic tests and validated clinical guidelines appear imperative.

Eosinophilic meningitis is the main but not the only clinical manifestation of angiostrongyliasis cantonensis [36]. Other symptoms include eosinophilic meningoencephalitis [37], eosinophilic radiculomyelitis [38], ocular angiostrongyliasis [39], as well as nasal [40] or pulmonary infections [41],[42]. Further, most angiostrongyliasis infections are self-limiting and recovery without sequelae is the norm [11],[19],[35]. However, ensuing encephalitis might be fatal [36]. All but one of the 33 patients described here developed eosinophilic meningitis and one patient suffered from eosinophilic radiculomyelitis. Severe headache and high intracranial pressure were often reported, and elevated eosinophil counts in blood and/or CSF were important characteristics [43]. Few studies describe the dynamics of eosinophils in peripheral blood [17],[19], and no complete data are available showing the co-dynamics of counts in peripheral blood and CSF which could support diagnosis and facilitate treatment evaluation. Indeed, these changes are difficult to generalize because many factors including age, severity and duration of the infection and treatment can affect eosinophil cell numbers. In the present survey, eosinophil counts were elevated in 84.8% of the participants, either in peripheral blood and/or in CSF, but the agreement between peripheral blood and CSF when patients were not treated was only 61.1%. Although the sample was small, the available data suggested that very young and elder patients were more likely to exhibit elevated counts in the CSF rather than in peripheral blood, and that eosinophil cell counts in the CSF returned to normal more quickly than those in blood.

A combination therapy with anthelminthic drugs and corticosteroids is recommended for the treatment of eosinophilic meningitis [17],[44],[45] although the role of anthelminthics in improving manifestations is still unclear [36],[46]. Presumably, anthelminthic drugs kill causative worm larvae and the corticosteroids suppress hypersensitivity reactions triggered by antigens released from the worms. Thus, anthelminthic treatment alone tends to exacerbate neurologic symptoms [6],[47], and corticosteroid treatment alone improves symptoms but fails to cure patients, resulting in relapses [9], [48]–[50]. In the present study, 26 patients were given a combination treatment resulting in significantly improved conditions. Dramatic drops in eosinophil counts over the course of the combination treatment were noted in those seven patients for whom complete data were available. However, no reliable conclusion was drawn due to small sample size and few repeated measurements of eosinophil counts.

Unlike most food-borne viral and bacterial diseases, food-borne helminthiasis usually has a long and variable incubation period, resulting in a tempo-spatially dispersed patient population. Incubation periods ranged from 3 to 50 days with a mean of 16 days in this outbreak. Moreover, patients did not appear to have consumed contaminated food at the same time, further spreading the temporal distribution of cases and masking the onset of the epidemic. This also made it difficult to determine the population potentially exposed to *A. cantonensis* infected food. It can thus be suspected that the 41 cases detected through retrospective analysis of patient data as well as the hospital-based surveillance system might represent but a fraction of all patients and thus not accurately reflect the afflicted population and the full range of clinical presentations and disease progression. This underscored the need to pay due attention to apparently isolated cases since they might signal the onset of a protracted epidemic. Hospital-based surveillance might be an effective approach to reveal unfolding angiostrongyliasis epidemics in areas where snails are commonly eaten.

In conclusion, the reported epidemic of angiostrongyliasis in Dali highlights the risks of food-borne diseases in hitherto non-endemic areas through food trade. It appears urgent to develop reliable diagnostic tools and establish sound diagnostic criteria and treatment schedules for angiostrongyliasis. Robust health education should be initiated and hospital-based surveillance established in regions where snails, particularly *P. canaliculata* and *A. fulica*, are popular.

## Supporting Information

Table S1 The diagnoses and clinical evidence of 33 patients.
(0.04 MB XLS)
